# 免疫靶向治疗在复发/难治性急性B淋巴细胞白血病患者中的疗效分析

**DOI:** 10.3760/cma.j.issn.0253-2727.2022.11.011

**Published:** 2022-11

**Authors:** 燕 苏, 慎 包, 玉萍 魏, 丽君 宋, 毅苗 薛, 旭东 魏, 永平 宋, 青松 尹

**Affiliations:** 1 河南省肿瘤医院、郑州大学附属肿瘤医院，郑州 450008 Department of Hematology, Henan Cancer Hospital; The Affiliated Cancer Hospital of Zhengzhou University, Zhengzhou 450008, China; 2 宁夏回族自治区人民医院、宁夏医科大学第三临床医学院，银川 750002 People's Hospital of Ningxia Hui Autonomous Region, Third Clinical Medical College of Ningxia Medical University, Yinchuan 750002, China

**Keywords:** 白血病，B淋巴细胞，急性, CAR-T细胞, Blinatumomab, Leukemia, B-cell, acute, CAR-T cell, Blinatumomab

## Abstract

**目的:**

比较复发/难治（R/R）急性B淋巴细胞白血病（B-ALL）患者以常规化疗和免疫靶向治疗为挽救治疗的疗效。

**方法:**

回顾性分析2008年1月至2020年7月郑州大学附属肿瘤医院收治的212例R/R B-ALL患者的临床资料，分析传统化疗与针对CD19嵌合抗原受体T细胞（CAR-T细胞）和CD3CD19双特异性抗体（blinatumomab，简称双特异性抗体）的免疫靶向治疗的缓解率及生存差异，并分析其相关影响因素。

**结果:**

CAR-T细胞治疗组的完全缓解（CR）率为80.4％（45/56），双特异性抗体治疗组的CR率为62.5％（5/8），传统化疗组的CR率为38.6％（56/145），三种方法挽救治疗的CR率差异有统计学意义（*P*<0.001）。CAR-T细胞治疗组患者的1年总生存（OS）率和无进展生存（PFS）率分别为41.5％和30.1％，显著高于传统化疗组的10.3％和9.7％（*P*值均<0.001）。双特异性抗体治疗组的1年OS率和PFS率分别为14.3％和14.6％，均高于传统化疗组（*P*值分别为0.018和0.046）。CAR-T细胞治疗达CR后桥接异基因造血干细胞移植（allo-HSCT）患者的中位OS及PFS时间（分别为18.5个月和17个月）较未接受allo-HSCT患者（分别为8个月和4个月）显著延长（*P*值均<0.05）。双特异性抗体治疗后桥接allo-HSCT患者的中位OS及PFS时间（分别为13个月和11个月）较未接受allo-HSCT患者（分别为9.5个月和6个月）延长。CAR-T细胞治疗的细胞因子释放综合征（CRS）的发生率为89.8％，其中≥3级CRS发生率为30.2％，双特异性抗体治疗组的CRS发生率为37.5％，且均为1级。

**结论:**

CD19 CAR-T细胞及CD3CD19双特异性抗体治疗R/R B-ALL患者的缓解率及生存率均显著优于传统化疗。

## Abstract (en)

**Objective:**

Comparison of conventional chemotherapy and immunotargeted therapy efficacy in patients with relapsed/refractory（R/R）acute B cell leukemia（B-ALL）.

**Methods:**

The clinical data of 212 patients with R/R B-ALL in the Affiliaed Cancer Hospital of Zhengzhou University from January 2008 to July 2020 were analyzed retrospectively to compare the response rate and survival time difference between conventional chemotherapy and immunotargeted therapies（antiCD19 CAR-T and CD3CD19 bi-specific antibody blinatumomab）, and to explore the related factors affecting prognosis.

**Results:**

The CR rate of patients with R/R B-ALL treated with anti-CD19 CAR-T cells was 80.4％, patients treated with blinatumomab was 62.5％, and patients treated with chemotherapy was 38.6％. There was significant difference in the CR rate among the three therapies（*P*<0.001）. CAR-T cells 1-year OS rate was 41.5％, which was significantly higher than that of the chemotherapy group（10.3％）（*P*<0.001）. The 1-year PFS rate of CAR-T cells（30.1％）was also significantly higher than that of the chemotherapy group（9.7％）（*P*<0.001）. The median OS of patients with bridging allo-HSCT after CR treatment by CAR-T cells was 18.5 months, which was higher than that of patients without allo-HSCT（8 months）（*P*＝0.027）. The median PFS of patients with allo-HSCT was 17 months, which was higher than that of patients without allo-HSCT（4 months）（*P*＝0.001）. The 1-year OS rate of patients treated with blinatumomab was 14.3％, which was higher than that of the chemotherapy group（10.3％）（*P*＝0.018）. The 1-year PFS rate（14.6％）was also higher than that of the chemotherapy group（9.7％）（*P*＝0.046）. The median OS and median PFS of patients with bridging allo-HSCT were 13 and 11 months, respectively, which was higher than that of patients without allo-HSCT（9.5 and 6 months）. The cytokine release syndrome（CRS）incidence in patients with R/R B-ALL treated with anti-CAR-T cells was 89.8％. Grades 3–4, grade 2, and grade 1 CRS were experienced by 30.2％, 11.3％ and 58.5％ patients, respectively. Only three patients（37.5％）with blinatumomab developed CRS, all of which were grade 1.

**Conclusion:**

The response rate and survival rate of patients with R/R B-ALL treated with CD19 CAR-T cells and blinatumomab were significantly better than those treated with conventional chemotherapy.

B细胞急性淋巴细胞白血病（B-ALL）是一种好发于儿童的克隆性增殖性血液系统恶性疾病[1]。近年来，儿童样化疗方案的应用极大地改善了青少年及年轻成人B-ALL患者的疗效。但仍有约65％ 的成人和约20％ 的儿童患者呈现难治或复发，常规化疗挽救诱导治疗的完全缓解（CR）率仅20％～30％，中位生存时间仅2～4个月[2]。嵌合抗原受体T细胞（CAR-T细胞）疗法是新型肿瘤免疫疗法，在复发/难治（R/R）B-ALL患者中取得了突出的疗效，CR率高达90％左右[3]–[4]。CD3CD19双特异性抗体主要通过激活内源性CD3^+^ T细胞杀伤CD19^+^的B-ALL细胞[5]，CR率为43％～69％[6]–[7]。本研究回顾性分析化疗及细胞免疫靶向治疗（抗CD19 CAR-T细胞和CD3CD19双特异性抗体）在R/R B-ALL患者中的疗效及生存差异。

## 病例与方法

1. 临床资料：回顾性分析2008年1月至2020年7月就诊于郑州大学附属肿瘤医院的212例R/R B-ALL患者，所有患者均符合R/R B-ALL的诊断标准[8]，排除其他血液系统疾病，收集患者的相关病例资料。

2. 治疗和分组方法：患者根据治疗方案分为3组：应用传统化疗方案治疗的患者145例，为传统化疗组；应用抗CD19 CAR-T细胞治疗的患者59例，为CAR-T细胞组；应用CD3CD19双特异性抗体（blinatumomab）治疗的患者有8例，为双特异性抗体组。传统化疗组的治疗方案包括：Hyper-CVAD/MA（A/B）方案（环磷酰胺+长春新碱+阿霉素+地塞米松、甲氨蝶呤+阿糖胞苷）、VMCP方案（长春新碱+6-巯基嘌呤+环磷酰胺+泼尼松）、FLAG方案（氟达拉滨+阿糖胞苷+G-CSF）、甲氨蝶呤+阿糖胞苷方案、COAD方案（环磷酰胺+长春新碱+阿糖胞苷+地塞米松）、CAM方案（环磷酰胺+阿糖胞苷+6-巯基嘌呤）、CAG+P+L-ASP方案（阿糖胞苷+阿克拉霉素+G-CSF+泼尼松+左旋门冬酰胺酶）等，Ph阳性的患者联合酪氨酸激酶抑制剂（TKI）；CAR-T细胞组：先采集患者外周血淋巴细胞（PBL）制备CD19 CAR-T细胞，CAR-T细胞回输前予以FC方案预处理：氟达拉滨25～30 mg/m^2^ −4～−2 d及环磷酰胺400～500 mg/m^2^ −4～−3 d，预处理后48 h，于第0天回输CD19 CAR-T细胞（回输细胞数为1×10^5^/kg～1.5×10^6^/kg）。回输后第14～28天进行骨髓细胞形态学检测评估疗效。双特异性抗体组：blinatumomab第1疗程9 µg第1～7天，28 µg第8～28天；第2疗程及以后28 µg第1～28天，包括2个疗程诱导缓解治疗和3个疗程巩固治疗，每个疗程包括4周持续静脉滴注给药和2周停药间歇期。

3. 疗效判断标准及不良反应评价标准：疗效包括形态学完全缓解（CR）、部分缓解（PR）、微小残留病（MRD）阴性CR、疾病进展（PD）与疾病复发[8]。按照细胞因子释放综合征（CRS）的诊断标准[9]，将患者治疗后并发的CRS分为1～5级。

4. 生存评价及随访：总生存（OS）时间指患者难治或复发后开始应用CAR-T细胞、双特异性抗体或化疗的时间至患者任何原因死亡或末次随访时间；无进展生存（PFS）时间为患者难治或复发后开始应用CAR-T细胞、双特异性抗体或化疗的时间至再次复发或任何原因导致死亡的时间（失访患者为末次随访时间）。所有病例通过电话联系、查阅近期复查信息等方法进行随访。随访截止日期为2020年9月。

5. 统计学处理：应用SPSS 24.0软件及Graphpad prism7.0软件进行统计学分析，组间率的比较采用卡方检验或Fisher精确概率法，使用Kaplan-Meier法进行生存分析并绘制生存曲线，各组之间生存率的比较采用Log-rank检验；*P*<0.05为差异有统计学意义。

## 结果

1. 一般临床资料：212例R/R B-ALL患者中男112例，女100例，男女之比为1.1∶1；中位年龄为21（1～72）岁。212例R/R B-ALL患者的临床特征详见表1。三组患者在性别、年龄、初诊时的血常规、初诊时骨髓中原始细胞比例、染色体是否异常、是否伴髓系抗原表达、诱导治疗后是否CR、是否有髓外侵犯、是否行异基因造血干细胞移植（allo-HSCT）等方面差异均无统计学意义（*P*值均>0.05）。

**表1:** 212例复发/难治（R/R）急性B淋巴细胞白血病患者的临床特征［例（％）］

临床特征	患者总体	CAR-T细胞组（59例）	传统化疗组（145例）	双特异性抗体组（8例）
年龄				
<30岁	135（63.7）	44（74.6）	90（62.1）	1（12.5）
≥30岁	77（36.3）	15（10.3）	55（37.9）	7（87.5）
性别				
男	112（52.8）	27（45.8）	82（56.6）	3（37.5）
女	100（47.2）	32（54.2）	63（43.4）	5（62.5）
初诊骨髓中原始幼稚淋巴细胞			
<90%	107（50.5）	30（50.8）	71（49.0）	6（75.0）
≥90%	90（42.5）	22（37.3）	66（45.5）	2（25.0）
缺失	15（7.0）	7（11.9）	8（5.5）	0（0）
诱导治疗后是否CR				
是	176（83.0）	50（84.7）	120（82.8）	6（75.0）
否	36（17.0）	9（15.3）	25（17.2）	2（25.0）
髓外侵犯				
有	50（23.6）	12（20.3）	37（25.5）	1（12.5）
无	162（76.4）	47（79.7）	108（74.5）	7（87.5）
初诊WBC				
<30×10^9^/L	109（51.4）	34（57.6）	70（48.3）	5（62.5）
≥30×10^9^/L	72（34.0）	16（27.1）	54（37.2）	2（25.0）
缺失	31（14.6）	9（15.3）	21（14.5）	1（12.5）
初诊HGB				
<90 g/L	105（49.5）	34（57.6）	67（46.2）	4（50.0）
≥90 g/L	69（32.5）	16（27.1）	50（34.5）	3（37.5）
缺失	38（17.9）	9（15.3）	28（19.3）	1（12.5）
初诊PLT				
<100×10^9^/L	142（67.0）	43（72.8）	95（65.5）	4（50.0）
≥100×10^9^/L	35（16.5）	8（13.6）	24（16.6）	3（37.5）
缺失	35（16.5）	8（13.6）	26（17.9）	1（12.5）
染色体				
异常	97（45.7）	25（42.4）	70（48.3）	2（25.0）
正常	47（22.2）	14（23.7）	30（20.7）	3（37.5）
未检测	68（32.1）	20（33.9）	45（31.0）	3（37.5）
伴髓系抗原表达				
是	108（50.9）	23（39.0）	79（54.5）	6（75.0）
否	72（34.0）	24（40.7）	46（31.7）	2（25.0）
缺失	32（15.1）	12（20.3）	20（13.8）	0（0）
allo-HSCT				
是	49（23.1）	18（30.5）	28（19.3）	3（37.5）
否	163（76.9）	41（69.5）	117（80.7）	5（62.5）

注 CR：完全缓解；allo-HSCT：异基因造血干细胞移植

2. 疗效及不良反应：59例CAR-T细胞组患者中，可评估疗效者56例，CR率为80.4％（45/56），其中MRD转阴率为86.7％（39/45）；双特异性抗体组CR率为62.5％（5/8），MRD转阴率60％（3/5）；传统化疗组CR率为38.6％（56/145），MRD转阴率33.9％（19/56）；三种治疗方法的CR率差异有统计学意义（*P*<0.001）。CAR-T细胞组患者的缓解率明显高于传统化疗组（*P*<0.001），稍高于双特异性抗体组，但差异未显示出统计学意义（*P*＝0.493），双特异性抗体组的CR率高于传统化疗组，差异无统计学意义（*P*＝0.331），可能与样本例数少有关。

59例接受抗CD19 CAR-T细胞的患者中，3例早期死亡，其中2例分别于CAR-T细胞回输后第6天和第14天因突发急性心脏事件死亡，1例因重症感染死亡。53例（89.8％）患者发生1～4级CRS，≥3级CRS的患者占30.2％（16/53），其中2例患者为中枢神经系统CRS，3级以下CRS的发生率为69.8％（37/53），其中2级CRS发生率为11.3％（6/53），1级CRS发生率为58.5％（31/53）。8例CD3CD19双特异性抗体治疗的患者中共3例（37.5％）发生了CRS，且均为1级。轻度CRS采用非甾体类消炎药对症治疗，≥3级CRS采用IL-6单抗（tocilizumab）及糖皮质激素等对症支持治疗，无CRS相关死亡。

3. 生存分析：212例R/R B-ALL患者中，CAR-T细胞组患者的中位OS和PFS时间分别为10个月和5个月，双特异性抗体组为12个月和7个月，传统化疗组为5个月和3个月（图1）。CAR-T细胞组患者1年OS率和PFS率分别为41.5％和30.1％，高于传统化疗组的10.3％和9.7％（*P*值均<0.001）。双特异性抗体治疗患者1年的OS率和PFS率分别为14.3％和14.6％，均高于传统化疗组（*P*值分别为0.018和0.046）。

**图1:**
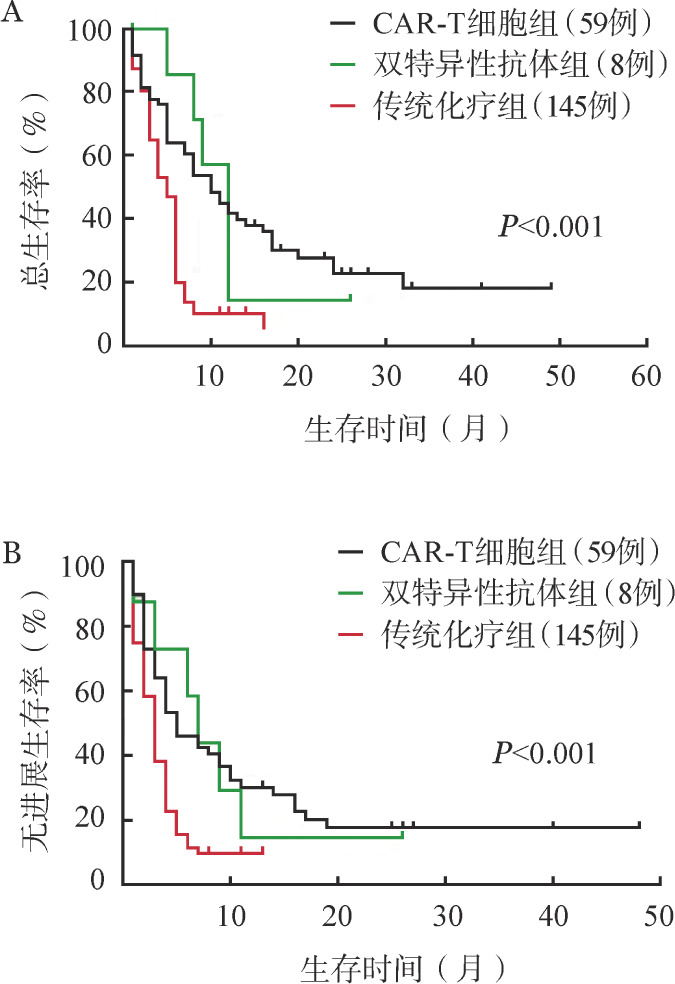
传统化疗、CAR-T细胞及CD3CD19双特异性抗体治疗对患者总生存（A）和无进展生存（B）的影响 注 CAR-T细胞：嵌合抗原受体T细胞

CAR-T细胞组59例患者中18例接受allo-HSCT，其中有6例为allo-HSCT后复发选择CAR-T细胞治疗，余12例均为CAR-T细胞治疗达CR后桥接allo-HSCT。桥接allo-HSCT患者中位OS和PFS时间分别为18.5个月和17个月，未接受allo-HSCT患者中位OS和PFS时间分别为8个月和4个月（*P*值分别为0.027和<0.001）；桥接allo-HSCT患者1年OS率和PFS率（分别为83.3％和81.5％）均明显高于未移植患者（分别为31.7％和12.9％）（*P*值均<0.05）（图2）。8例接受双特异性抗体治疗的患者中3例双特异性抗体治疗后桥接allo-HSCT，桥接allo-HSCT患者的OS时间分别为13、13、27个月，PFS时间分别为9、11、26个月，未接受allo-HSCT患者的中位OS和中位PFS时间分别为9.5（1～12）个月和6（1～7）个月。

**图2:**
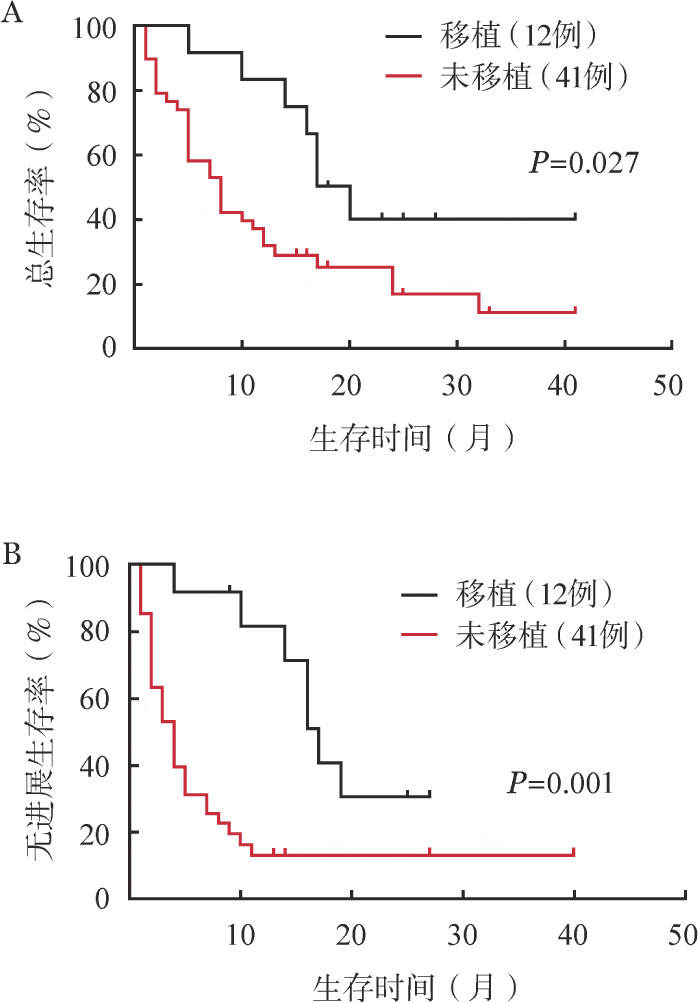
CAR-T细胞治疗后桥接allo-HSCT对患者总生存（A）和无进展生存（B）的影响 注 CAR-T细胞：嵌合抗原受体T细胞；allo-HSCT：异基因造血干细胞移植

## 讨论

随着对B-ALL生物学特性的深入研究及治疗方案的改进，儿童及青少年B-ALL的疗效和预后得到极大改善，但成年患者的长期存活率仅35％～45％，预后差[2]。B-ALL治疗失败的主要原因是原发耐药和复发，再次诱导的缓解率低，5年生存率低于15％，亟待寻找提高R/R患者疗效的治疗措施。目前基于肿瘤相关抗原表达的免疫治疗明显提高了R/R B-ALL患者的疗效，如抗CD22单体（Ino）[10]，靶向CD19的CD3CD19双特异性抗体[6]–[7]及抗CD19 CAR-T细胞[11]–[13]。

Blinatumomab是CD3及CD19单抗通过双特异性T细胞衔接器（BiTE）耦连的单链结构抗体，多个研究报道blinatumomab单药治疗R/R ALL的CR率为43％～69％，中位OS时间为6.1～13个月[6]–[7],[14]，明显优于传统化疗，且肿瘤负荷越低疗效越佳，对MRD^+^者疗效更佳[15]。本研究中blinatumomab组治疗的CR率为62.5％（5/8），MRD转阴率60％（3/5）；中位OS时间为12个月，中位PFS时间为7个月，与文献报道基本一致。研究显示blinatumomab治疗显著增加桥接allo-HSCT的机会，桥接HSCT可进一步改善R/R ALL患者的生存[15]。本研究中应用blinatumomab治疗后桥接allo-HSCT患者的中位OS和PFS时间分别为13和11个月，显著长于未移植患者的9.5和6个月，与文献报道一致[15]。但由于本研究接受blinatumomab治疗患者例数较少，仍有待扩大样本量进一步证实。

自2011年June教授首次报道CD19 CAR-T细胞成功治疗1例R/R B-ALL患儿以来，后续报道显示其用于R/R ALL患者CR率达90％左右[11]–[13]。本研究中CAR-T细胞组的疗效显著优于传统化疗，CR率分别为80.4％和38.6％。CAR-T细胞组患者的1年OS率和PFS率分别为41.5％和30.1％，高于化疗组的10.3％和9.7％，与文献报道基本一致[11]–[13]。但CAR-T细胞治疗后3～6个月复发率达60％～70％[16]。因此，亟待寻找CAR-T细胞治疗后的巩固维持治疗，以提高患者的长期生存率。研究显示，CAR-T细胞后是否接受allo-HSCT是影响患者无病生存（DFS）和OS的独立预后因素[17]–[20]。一项纳入110例具有高危因素的R/R B-ALL患者的研究报道，抗CD19 CAR-T细胞治疗后桥接allo-HSCT患者的1年OS率和PFS率（分别为79.1％和76.9％）明显高于单纯CAR-T细胞治疗的患者（分别为32％和11.6％）[18]。本研究发现CAR-T细胞治疗达CR后桥接allo-HSCT患者的1年OS率和PFS率（分别为83.3％和81.5％），均明显高于未移植患者（分别为31.7％和12.9％），与报道一致，提示CAR-T细胞治疗达CR后桥接allo-HSCT可显著延长患者的OS及PFS时间。此外，研究发现CAR-T细胞的结构、CD4^+^/CD8^+^ T细胞的比率、负载CAR的T细胞类型等均影响CAR-T细胞的持久性[17]。CAR-T细胞治疗前的疾病负荷[15],[21]、治疗后MRD状态[17]、相关突变基因（如TP53突变）[18]、淋巴清除[17]、实验室相关指标（如乳酸脱氢酶浓度和血小板计数）[17]等均影响治疗后长期疗效。

CRS和CAR-T细胞相关脑病综合征（CRES）是CAR-T细胞治疗常见的毒性反应，严重CRS和CRES发生率分别为23％～49％和13％～50％，其发生与预处理方案、CAR-T细胞输注量和CAR结构可能有关[22]。本研究中应用CAR-T细胞治疗患者≥3级CRS的发生率为30.2％，与报道一致[22]，CRES的发生率约3.8％，略低于研究报道[22]。本研究中应用双特异性抗体治疗的患者仅37.5％发生CRS，且均为1级，与报道一致[13]–[14]。对于≥3级CRS及CRES应及时采用IL-6单抗治疗，尚未发现其对CAR-T细胞疗效的影响[22]。对tocilizumab疗效不佳及严重CRS推荐短疗程的糖皮质激素治疗[22]。

总之，标准化疗方案对R/R B-ALL患者的疗效有限，靶向CD19的CAR-T细胞及CD3CD19双特异性抗体治疗提高了R/R B-ALL的缓解率及生存时间，桥接allo-HSCT获益更多，但对老年体弱患者警惕CAR-T细胞的CRS。
